# Effects of RAS and SGLT2 inhibitors alone or in combination on end-stage kidney disease and/or all-cause death in patients with both diabetes and hypertension: a nationwide cohort study

**DOI:** 10.1186/s12933-025-02846-x

**Published:** 2025-07-14

**Authors:** Sangmo Hong, Kyungdo Han, Kyung-Soo Kim, Cheol-Young Park

**Affiliations:** 1 Department of Internal Medicine, College of Medicine, Guri Hospital, Hanyang University, Seoul, Republic of Korea; 2 Department of Statistics and Actuarial Science, Soongsil University, Seoul, Republic of Korea; 3 Department of Internal Medicine, CHA Bundang Medical Center, CHA University School of Medicine, Seongnam, Republic of Korea; 4 Division of Endocrinology and Metabolism, Department of Internal Medicine, Samsung Kangbuk Hospital, Sungkyunkwan University School of Medicine, Seoul, Republic of Korea

**Keywords:** End-stage kidney disease, Sodium-glucose cotransporter-2 inhibitors, Renin-angiotensin system inhibitors, Chronic kidney disease, Type 2 diabetes, Hypertension, All-cause mortality

## Abstract

**Background:**

Renin–angiotensin–aldosterone system (RAS) inhibitors and sodium-glucose cotransporter 2 (SGLT2) inhibitors are key treatments for diabetic kidney disease. However, their independent and combined effects on end-stage kidney disease (ESKD) and mortality remain unclear. This study evaluates their impact, alone or in combination, on ESKD and all-cause mortality in patients with diabetes and hypertension.

**Methods:**

A nationwide cohort study using the Korean National Health Database included 261,783 individuals with type 2 diabetes and hypertension (2015–2017). Participants were grouped into (1) no RAS or SGLT2 inhibitors (reference), (2) SGLT2 inhibitors alone, (3) RAS inhibitors alone, and (4) combination therapy. Cox regression models were used to estimate hazard ratios (HRs) for ESKD, mortality, and their composite.

**Results:**

Over 5.38 years, 2,674 (1.02%) developed ESKD and 20,866 (7.97%) died. Combination therapy showed the greatest risk reduction for composite outcomes [HR 0.68, 95% confidence interval (CI) 0.56–0.82] and mortality (HR 0.68, 95% CI 0.56–0.83). SGLT2 inhibitors alone reduced composite risk (HR 0.71, 95% CI 0.61–0.84) and mortality (HR 0.68, 95% CI 0.57–0.81). RAS inhibitors alone had modest effects (HR 0.96, 95% CI 0.93–0.98) on composite outcomes and mortality (HR 0.94, 95% CI 0.91–0.97). Notably, only combination therapy was associated with lower ESKD risk (HR 0.63, 95% CI 0.37–1.07), but this was not statistically significant. SGLT2 inhibitors consistently reduced ESKD and mortality, while RAS inhibitors were beneficial mainly in non-SGLT2 inhibitor users.

**Conclusion:**

Combination therapy may provide the greatest renal and survival benefit for diabetic patients with hypertension. SGLT2 inhibitors alone significantly reduced mortality, while RAS inhibitors alone had a modest impact.

**Graphical abstract:**

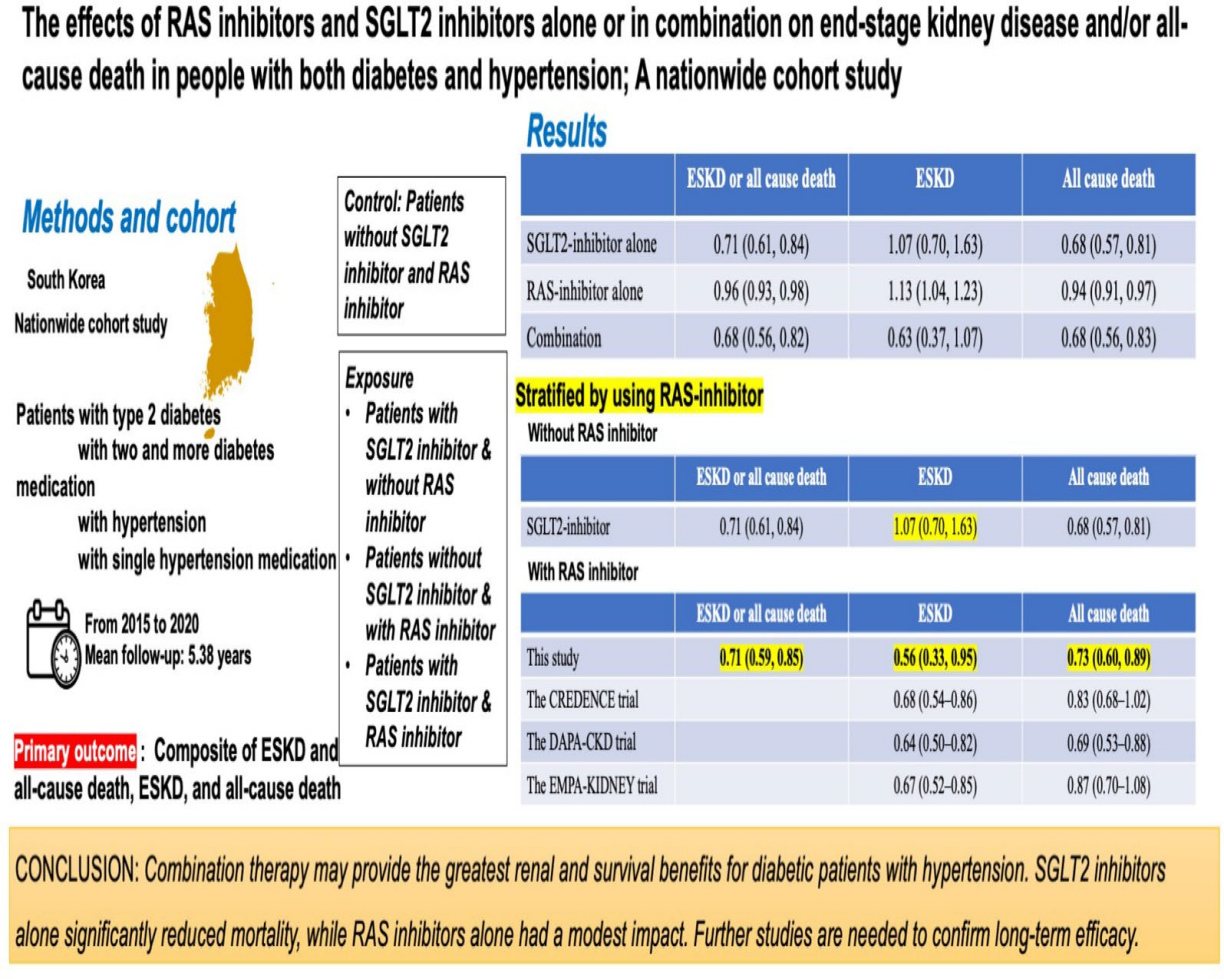

**Supplementary Information:**

The online version contains supplementary material available at 10.1186/s12933-025-02846-x.

## Background

Chronic kidney disease (CKD) is a prevalent, burdensome, and costly condition among individuals with diabetes [1, 2]. CKD is defined by structural or functional kidney abnormalities persisting for at least three months and has potential health consequences [2, 3]. CKD can advance to kidney failure requiring dialysis or kidney transplantation and is the primary cause of end-stage kidney disease (ESKD), with diabetes being the most common underlying factor [4, 5]. The development of diabetic kidney disease (DKD) involves the upregulation of the renin–angiotensin–aldosterone system (RAS) and alterations in renal hemodynamics with glomerular hypertension, ischemia, and oxidative stress [6–9]. However, the complete pathogenesis of DKD is not fully understood, and specific therapeutic targets have not been clearly identified. Patients with DKD frequently experience multimorbidity, including CKD progression, cardiovascular complications, and premature mortality [10, 11]. Therefore, a multidisciplinary treatment approach is essential, incorporating blood glucose management, blood pressure and lipid control using RAS inhibitors, weight management, dietary guidance, and smoking cessation counseling [2, 3, 7].

In addition to multidisciplinary treatments, sodium-glucose cotransporter 2 (SGLT2) inhibitors have been introduced as a new therapeutic option for DKD. Several studies analyzing the renal effects of SGLT2 inhibitors as secondary outcomes in large cardiovascular outcome trials involving individuals with type 2 diabetes have demonstrated the renal benefits of SGLT2 inhibitors [12–14]. Three recent large-scale clinical trials of SGLT2 inhibitors that focused on people with CKD and assessed primary kidney outcomes demonstrated clear [15–17]. All trials that evaluated the benefits of SGLT2 inhibition were performed in a population where more than 80% of individuals were being treated with an angiotensin-converting enzyme (ACE) inhibitor or angiotensin receptor blocker (ARB), in some trials up to maximum tolerated doses. However, there is a lack of research on whether the effects of RAS inhibitors and SGLT2 inhibitors are synergistic or independent, and whether similar benefits are observed with other antihypertensive agents. In this study, we analyzed the effects of RAS inhibitors and SGLT2 inhibitors alone or in combination on ESKD in people with both diabetes and hypertension using a large-scale population dataset from the National Health Information Database (NHID).

## Methods

### Study database

Our analysis utilized data from the NHID, a comprehensive public database in South Korea that includes healthcare utilization, health screening, sociodemographic, and mortality information for the entire population. Managed by the National Health Insurance Service (NHIS), the NHID was established in 2000 through the integration of 375 insurance associations and encompasses data spanning 2002–2017. It offers longitudinal information for 97% of the Korean population, with linkage to the National Death Registry and the national health screening program [18, 19]. The national health screening program, launched in 2009, includes a medical interview, a postural assessment, chest X-rays, blood tests (such as fasting glucose, lipid profile, liver function tests, and creatinine), urine tests, dental examinations, and other assessments. Approval for the study protocol was obtained from the Institutional Review Board of Gangbuk Samsung Hospital (KBSMC 2022-04-016), and the need for informed consent was waived by the board.

### Study participants

**Fig. 1:**
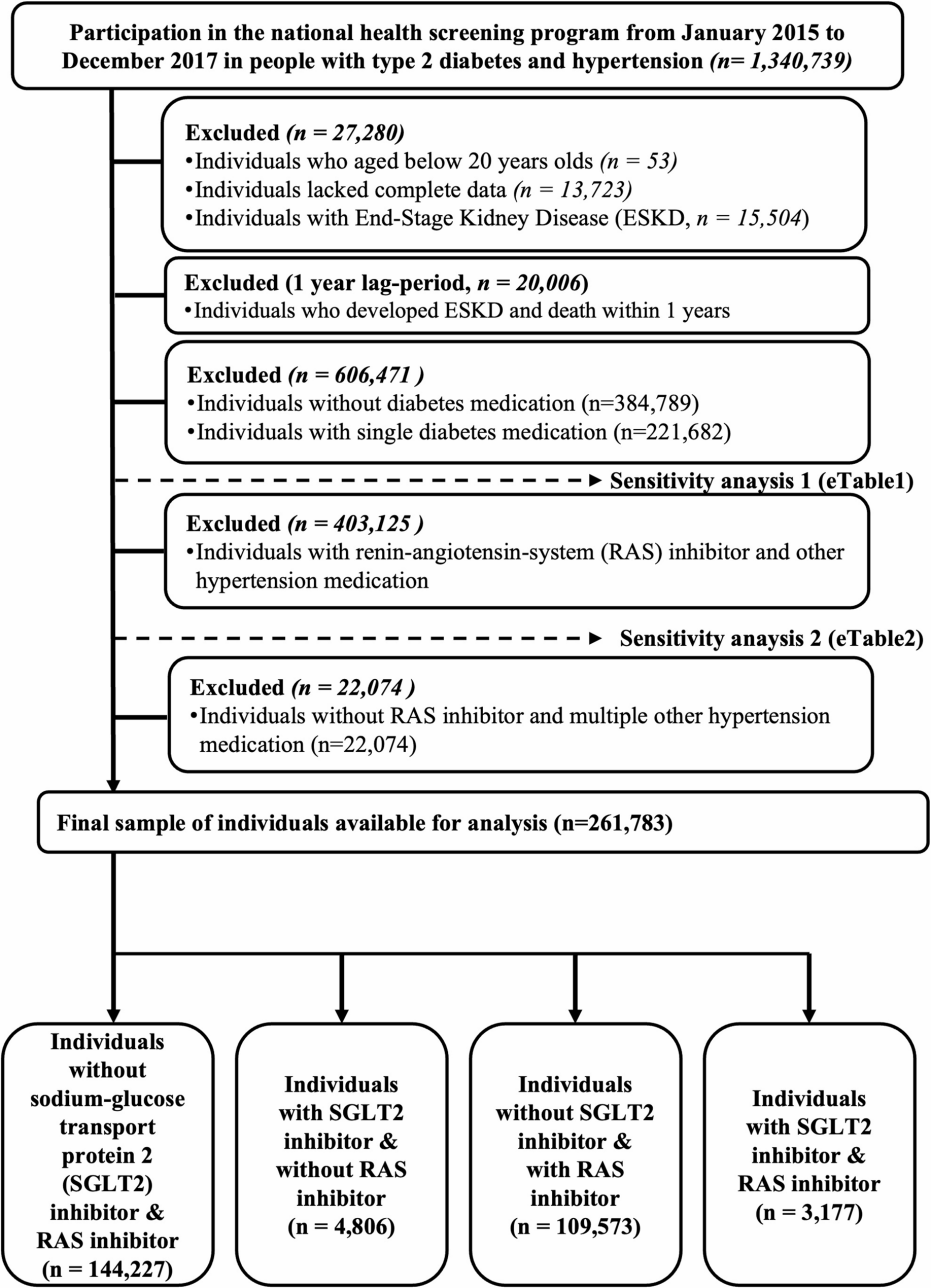
Flow chart of the study population

This national observational cohort study included 261,783 individuals. In total, 1,340,739 participants with type 2 diabetes and hypertension took part in the national health screening program between January 2015 and December 2017. Exclusions were made for 53 individuals under the age of 20, 13,723 individuals with incomplete data, and 15,504 individuals with a history of ESKD. To mitigate reverse causality, 20,006 individuals who developed ESKD or died within the first year (first-year lag period) were also excluded. Additionally, to reduce selection bias, we excluded 384,789 individuals not using diabetes medication; 221,682 individuals using a single diabetes medication; 403,125 individuals using RAS inhibitors along with other hypertension medications; and 22,074 individuals using multiple hypertension medications without RAS inhibitors (Fig. 1). Ultimately, 261,783 individuals were included in the analysis and were categorized into four groups: (1) 144,227 individuals not taking either SGLT2 inhibitors or RAS inhibitors; (2) 4,806 individuals taking SGLT2 inhibitors but not RAS inhibitors; (3) 109,573 individuals not taking SGLT2 inhibitors but on RAS inhibitors; and (4) 3,177 individuals on both SGLT2 inhibitors and RAS inhibitors.

### Definitions of the primary outcomes

The primary outcomes in our study were newly diagnosed ESKD, all-cause death, and the composite outcome of ESKD or all-cause death. ESKD was defined using a combination of ICD-10 codes (N18-19, Z49, Z94.0, Z99.2) and initiation of kidney replacement therapy and/or kidney transplantation. People undergoing kidney replacement therapy or kidney transplantation in Korea are classified as having a rare incurable disease and are eligible for full reimbursement of medical expenses related to dialysis. Kidney replacement therapy and/or kidney transplantation were defined based on rare incurable disease registry codes, including hemodialysis (V001), peritoneal dialysis (V003), and kidney transplantation (V005). The study population was followed from baseline to the date of ESKD, death, or until December 31, 2022, whichever came first.

### Comorbid metabolic diseases and clinical and laboratory measurements

Baseline comorbidities included hypertension (defined by ICD-10 codes I10 to I13 or I15, use of antihypertensive medications, systolic BP ≥ 140 mmHg, or diastolic BP ≥ 90 mmHg), type 2 diabetes (defined by ICD-10 codes E11 to E14, use of antidiabetic medications, or fasting glucose ≥ 126 mg/dL), and hyperlipidemia (defined by ICD-10 code E78, use of lipid-lowering agents, or serum total cholesterol ≥ 240 mg/dL). All participants completed a questionnaire covering medical history, tobacco and alcohol use, and exercise habits. Smoking status was classified as non-smoker, ex-smoker, or current smoker. Alcohol consumption was categorized as non-drinker, moderate drinker (< 30 g/day in men and  < 20 g/day in women), or heavy drinker (≥ 30 g/day in men and  ≥ 20 g/day in women). Regular exercise was defined as vigorous-intensity activities at least three times per week or moderate-intensity activities at least five times per week. Low socioeconomic status was identified as an income in the lowest 20% of the population. BMI was calculated as body weight (in kilograms) divided by height (in meters squared). Blood pressure (BP) was measured using the standard procedure with a sphygmomanometer after resting for more than 5 min. Blood samples were collected after overnight fasting. Serum glucose, total cholesterol, triglycerides (TG), high-density lipoprotein (HDL) cholesterol, and low-density lipoprotein (LDL) cholesterol were measured. We calculated glomerular filtration rate (GFR) using the four-variable Modification of Diet in Renal Disease Study equation [20].

### Data analyses

Baseline characteristics were analyzed using descriptive statistics. Categorical variables were described as a frequency or percentage. Continuous variables were described as the mean (± standard deviation [SD]) for normally distributed data and as the geometric mean and 95% confidence interval (CI) for data not normally distributed. We compared the baseline characteristics of the four groups based on the presence or absence of RAS inhibitors and SGLT2 inhibitors. Continuous variables were compared using one-way analysis of variance (ANOVA), while categorical variables were compared using the chi-square test. The follow-up duration of each group was obtained. The incidence rates of primary outcomes were estimated for each four over the total follow-up period. Incidence curves were estimated using the Kaplan–Meier method, and the log rank test was also conducted. All outcomes were analyzed by Cox proportional hazards regression analysis while controlling for baseline covariates. We deemed a two-tailed p-value less than 0.05 to be significant. Analyses were performed with SAS 9.4 (SAS Institute, Cary, NC, USA) and R program, version 3.4.1 (The R Foundation for Statistical Computing, Vienna, Austria, http://www.R-project.org).

## Results

### Baseline characteristics of subjects

Table 1 presents the baseline characteristics of the study population based on the prescription of RAS inhibitors and SGLT2 inhibitors. Individuals prescribed SGLT2 inhibitors had a greater proportion of current smokers and exhibited more pronounced metabolic abnormalities, including higher BMI, elevated fasting glucose levels, higher eGFR, and a higher prevalence of dyslipidemia compared to those not prescribed SGLT2 inhibitors. On the other hand, individuals prescribed RAS inhibitors were older, had a longer duration of comorbid diabetes and hypertension, higher triglyceride levels, and increased rates of proteinuria but lower systolic blood pressure and reduced eGFR compared to those not using RAS inhibitors, reflecting management of longstanding conditions.

**Table 1:** Baseline characteristics of the study population

Sodium-glucose co-transporter-2 inhibitors	Negative	Positive		Negative	Positive		*p* value
Renin-angiotensin-system inhibitors	Negative	Negative		Positive	Positive		
n	144,227	4806		109,573	3177		
Age, years	62.07 ± 11.62	54.11 ± 11.63		62.39 ± 10.76	55.41 ± 10.97		<.0001
Sex, male	85,145 (59.04)	2838 (59.05)		65,307 (59.6)	1816 (57.16)		0.0027
Body mass index, kg/m^2^	25.23 ± 3.45	27.13 ± 4.03		25.42 ± 3.43	27.33 ± 3.99		<.0001
Income, low 25%	33,340 (23.12)	1114 (23.18)		25,629 (23.39)	748 (23.54)		0.4251
*Smoking status*							<.0001
Never	82,679 (57.33)	2572 (53.52)		62,113 (56.69)	1730 (54.45)		
Former	32,844 (22.77)	1057 (21.99)		26,579 (24.26)	715 (22.51)		
Current	28,704 (19.9)	1177 (24.49)		20,881 (19.06)	732 (23.04)		
*Alcohol drinking*							<.0001
Non	89,973 (62.38)	2674 (55.64)		69,420 (63.36)	1851 (58.26)		
Mild	41,487 (28.77)	1602 (33.33)		31,113 (28.39)	1004 (31.6)		
Heavy	12,767 (8.85)	530 (11.03)		9040 (8.25)	322 (10.14)		
Regular exercise	30,382 (21.07)	910 (18.93)		23,957 (21.86)	603 (18.98)		<.0001
Systolic blood pressure, mmHg	139.0 ± 13.9	139.5 ± 13.4		133.3 ± 15.4	133.8 ± 15.1		<.0001
Diastolic blood pressure, mm Hg	84.0 ± 10.4	87.1 ± 10.2		80.0 ± 10.5	82.3 ± 10.8		<.0001
Fasting glucose, mg/dL	156.7 ± 53.9	168.5 ± 58.6		149.9 ± 50.5	159.9 ± 53.7		<.0001
Total cholesterol, mg/dL	181.76 ± 44.27	192.04 ± 48.34		172.36 ± 41.54	182.31 ± 49.35		<.0001
HDL cholesterol, mg/dL	50.6 ± 13.83	50.78 ± 12.75		49.5 ± 13.2	50.02 ± 12.59		<.0001
LDL cholesterol, mg/dL	99.4 ± 38.64	105.77 ± 41.52		92.14 ± 35.82	97.68 ± 42.59		<.0001
Triglycerides, mg/dL	140.86 (140.45–141.27)	158.95 (156.38–161.56)		135.94 (135.5–136.39)	155.7 (152.68–158.78)		<.0001
eGFR, mL/min/1.73 m^2^	90.04 ± 46.46	96.62 ± 47.74		86.2 ± 50.64	94.3 ± 60.38		<.0001
Proteinuria, positive	12,113 (8.4)	434 (9.03)		10,561 (9.64)	357 (11.24)		<.0001
*Diabetes duration (years)*							<.0001
< 5	52,957 (36.72)	2671 (55.58)		30,127 (27.49)	1440 (45.33)		
≥ 5, < 10	38,623 (26.78)	1027 (21.37)		30,252 (27.61)	707 (22.25)		
≥ 10	52,647 (36.5)	1108 (23.05)		49,194 (44.9)	1030 (32.42)		
*Diabetes medication*							
Insulin	15,647 (10.85)	462 (9.61)		14,619 (13.34)	414 (13.03)		<.0001
Metformin	138,801 (96.24)	4747 (98.77)		104,610 (95.47)	3130 (98.52)		<.0001
SU	87,263 (60.5)	2305 (47.96)		65,250 (59.55)	1531 (48.19)		<.0001
TZD	19,318 (13.39)	494 (10.28)		16,807 (15.34)	358 (11.27)		<.0001
AGI	5556 (3.85)	65 (1.35)		4072 (3.72)	53 (1.67)		<.0001
Meglitinides	738 (0.51)	18 (0.37)		692 (0.63)	7 (0.22)		<.0001
*Hypertension duration*							<.0001
< 5yrs	72,799 (50.48)	3170 (65.96)		34,891 (31.84)	1392 (43.81)		
< 10yrs	27,990 (19.41)	719 (14.96)		31,348 (28.61)	771 (24.27)		
≥ 10yrs	43,438 (30.12)	917 (19.08)		43,334 (39.55)	1014 (31.92)		
*Hypertension medication*							
BB	18,655 (12.93)	539 (11.22)		0 (0)	0 (0)		<.0001
CCB	42,195 (29.26)	760 (15.81)		0 (0)	0 (0)		<.0001
Diuretic	5017 (3.48)	156 (3.25)		0 (0)	0 (0)		<.0001
Dyslipidemia	92,369 (64.04)	3398 (70.7)		78,617 (71.75)	2485 (78.22)		<.0001
*The length of follow-up*							
Mean ± SD	5.33 ± 1.24	4.87 ± 0.85		5.47 ± 1.23	4.99 ± 0.86		<.0001
Median (Q1–Q3)	5.59 (4.79–6.15)	5.01 (4.22–5.41)		5.76 (5.05—6.24)	5.08 (4.45–5.53)		
*Index year*							<.0001
2015	57,220 (39.67)	449 (9.34)		50,144 (45.76)	363 (11.43)		
2016	54,944 (38.1)	2087 (43.42)		43,643 (39.83)	1627 (51.21)		
2017	32,063 (22.23)	2270 (47.23)		15,786 (14.41)	1187 (37.36)		

### Risk of ESKD/all-cause death composite, ESKD alone, and all-cause death alone

Table 2 provides an overview of the cumulative prevalence, annual incidence, and hazard ratios (HRs) for ESKD or all-cause death (composite), ESKD alone, and all-cause death across different groups. Over a median follow-up period of 5.38 years, 2,674 individuals (1.02%) developed ESKD, and 20,866 individuals (7.97%) died. eFigure 1 shows the cumulative incidence probabilities for three outcomes (ESKD or all-cause death, ESKD alone, and all-cause death) stratified according to the use of SGLT2 inhibitors and RAS inhibitors by Kaplan–Meier survival analysis. The incidence rate of all three outcomes (ESKD or all-cause death, ESKD, and all-cause death) in individuals with SGLT2 inhibitors was significantly lower than that in individuals without SGLT2 inhibitors (all log-rank, *p* < 0.001; eFig. 1). SGLT2 inhibitors, especially in combination with RAS inhibitors, appear to have a protective effect against progression to ESKD (eFig. 1B).

**Table 2:** Number, incidence rate, and hazard ratio of outcomes

	Number of patients	Number of events	Duration (person-years)	Rate*	Hazard ratio (95% confidence interval)		
					Unajusted	Model 1	Model 2
*ESKD or all-cause death*							
SGLT2-i (-) & RAS (-)	144,227	12,687	769,150	16.49	1 (ref.)	1 (ref.)	1 (ref.)
SGLT2-i ( +) & RAS-i (-)	4806	149	23,412	6.36	0.37 (0.31, 0.43)	0.69 (0.59, 0.81)	0.71 (0.61, 0.84)
SGLT2-i (-) & RAS-i ( +)	109,573	10,063	598,885	16.80	1.03 (1.00, 1.06)	1.05 (1.03, 1.08)	0.96 (0.93, 0.98)
SGLT2-i ( +) & RAS-i ( +)	3177	110	15,861	6.94	0.40 (0.33, 0.49)	0.73 (0.61, 0.88)	0.68 (0.56, 0.82)
*ESKD*							
SGLT2-i (-) & RAS (-)	144,227	1135	769,150	1.48	1 (ref.)	1 (ref.)	1 (ref.)
SGLT2-i ( +) & RAS-i (-)	4806	22	23,412	0.94	0.66 (0.43, 1.00)	0.73 (0.48, 1.11)	1.07 (0.70, 1.63)
SGLT2-i (-) & RAS-i ( +)	109,573	1503	598,885	2.51	1.69 (1.56, 1.83)	1.65 (1.52, 1.78)	1.13 (1.04, 1.23)
SGLT2-i ( +) & RAS-i ( +)	3177	14	15,861	0.88	0.61 (0.36, 1.04)	0.66 (0.39, 1.11)	0.63 (0.37, 1.07)
*All-cause death*							
SGLT2-i (-) & RAS (-)	144,227	11,785	771,448	15.28	1 (ref.)	1 (ref.)	1 (ref.)
SGLT2-i ( +) & RAS-i (-)	4806	127	23,459	5.41	0.33 (0.28, 0.40)	0.68 (0.57, 0.80)	0.68 (0.57, 0.81)
SGLT2-i (-) & RAS-i ( +)	109,573	8857	602,146	14.71	0.97 (0.95, 1.00)	1.01 (0.98, 1.04)	0.94 (0.91, 0.97)
SGLT2-i ( +) & RAS-i ( +)	3177	97	15,880	6.11	0.38 (0.31, 0.46)	0.74 (0.61, 0.90)	0.68 (0.56, 0.83)

*Rate: events per 1000 person-years

Model 1: adjusted by age and sex

Model 2: adjusted by age, sex, household income, smoking status, alcohol drinking, regular exercise, body mass index, systolic blood pressure, glucose, eGFR, duration of diabetes, duration of hypertension, diabetes medication, and hypertension medication

#### Risk of ESKD or all-cause death

**Fig. 2:**
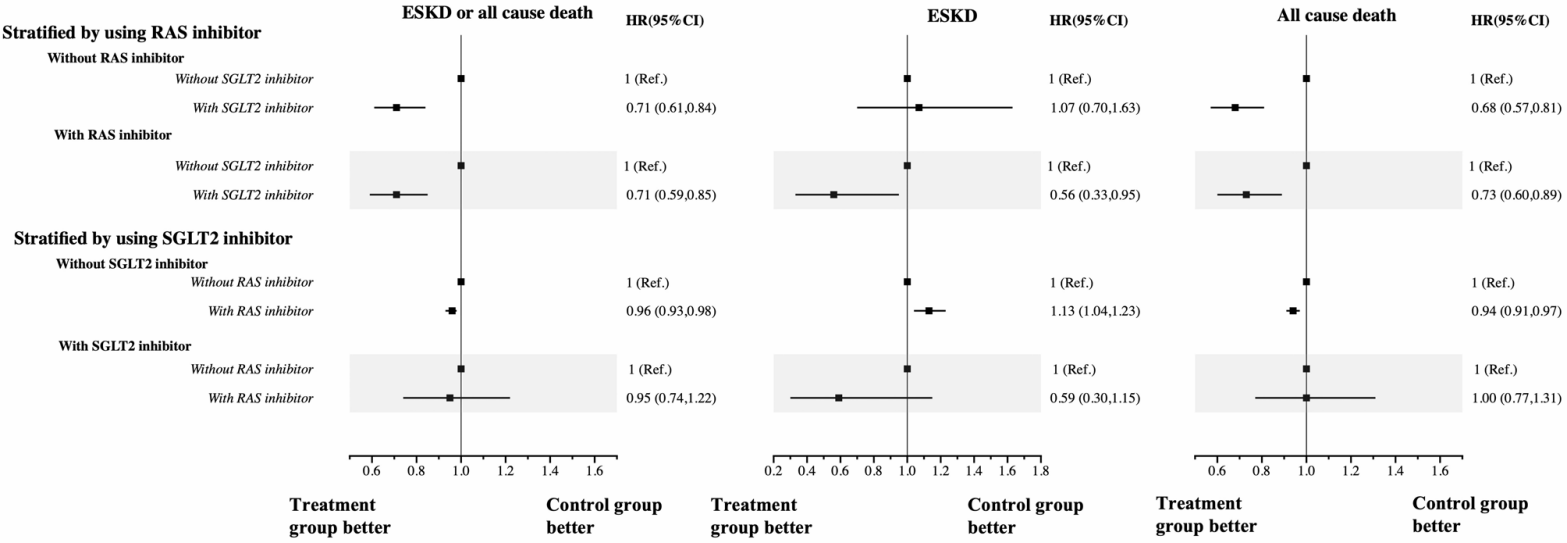
The hazard ratios for end-stage kidney disease (ESKD) and/or all-cause death were assessed by comparing patients treated with RAS inhibitors or SGLT2 inhibitors to the control group, stratified based on the use of RAS inhibitors or SGLT2 inhibitors

The reference group (without SGLT2 inhibitors or RAS inhibitors) had an incidence rate of 16.49 per 1,000 person-years. Individuals in the SGLT2-i alone group (using SGLT2 inhibitors but not RAS inhibitors) had a significantly lower incidence rate of 6.36 per 1,000 person-years, along with reduced risk of ESKD or all-cause death (HR 0.71, 95% CI 0.61–0.84; Table 2). Those in the RAS-i alone group (using RAS inhibitors but not SGLT2 inhibitors) had a higher incidence rate of 16.80 per 1,000 person-years and lower risk (HR 0.96, 95% CI 0.93–0.98; Table 2). Individuals in the combination group (using both SGLT2 and RAS inhibitors) had an incidence rate of 6.94 per 1,000 person-years and a significantly lower risk of ESKD or all-cause death (HR 0.68, 95% CI 0.56–0.82; Table 2). When analyzed separately through stratification, SGLT2 inhibitors were consistently associated with reduced risk of ESKD or all-cause death, whereas RAS inhibitors were only linked to reduced risk in individuals without SGLT2 inhibitors (Fig. 2). eFigure 2 shows the results of the subgroup analyses based on baseline characteristics. SGLT2-i and RAS-i combination therapy is generally associated with a reduced risk of ESKD or all-cause death across most subgroups (eFig. 2A). However, patients with longer hypertension duration (*p* < 0.001), those not using insulin (*p* = 0.002), and those with proteinuria (*p* = 0.003) appear to benefit more from SGLT2-i and RAS-i combination therapy (eFig. 2A). Also, patients with dyslipidemia appear to benefit more from SGLT2-i therapy (*p* = 0.001).

#### Risk of ESKD

The reference group had an incidence rate of 1.48 per 1,000 person-years. Individuals in the SGLT2-i alone group had a lower incidence rate of 0.94 per 1,000 person-years, but the adjusted hazard ratio was not statistically significant (HR 1.07, 95% CI 0.70–1.63, Table 2). Those in the RAS-i alone group had a higher incidence rate of 2.51 per 1,000 person-years and increased risk (HR 1.13, 95% CI 1.04–1.23; Table 2). Individuals in the combination group had the lowest incidence rate of 0.88 per 1,000 person-years and lower risk of ESKD (HR 0.63, 95% CI 0.37–1.07; Table 2), but this difference was not statistically significant. When analyzed separately through stratification, SGLT2-i was associated with reduced risk of ESKD only in those on RAS-i (HR 0.56, 95% CI 0.33–0.95). RAS-i was associated with reduced risk of ESKD only in those also on SGLT2-i (HR 0.59, 95% CI 0.30–11.5), but that was not significant (Fig. 2). eFigure 2 shows the results of the subgroup analyses based on baseline characteristics. SGLT2-i and RAS-i combination therapy was generally associated with a reduced risk of ESKD across most subgroups (eFig. 2B). However, patients 65 years or older (< 0.001), with longer diabetes duration (*p* = 0.046), and with longer hypertension duration (*p* < 0.001) appear to benefit more from SGLT2-i and RAS-i combination therapy (eFig. 2B).

#### Risk of all-cause death

The reference group had an incidence rate of 15.28 per 1,000 person-years. Individuals in the SGLT2-i alone group had the lowest incidence rate of 5.41 per 1,000 person-years along with reduced risk of all-cause death (HR 0.68, 95% CI 0.57–0.81; Table 2). Those in the RAS-i alone group had an incidence rate of 14.71 per 1,000 person-years and lower risk (HR 0.94, 95% CI 0.91–0.97; Table 2). Individuals in the combination group had a lower incidence rate of 6.11 per 1,000 person-years and significantly lower risk of ESKD (HR 0.68, 95% CI 0.56–0.83; Table 2). When analyzed separately through stratification, SGLT2 inhibitors were consistently associated with reduced risk of all-cause death, whereas RAS inhibitors were linked to lower risk only in those not using SGLT2-i (Fig. 2). eFigure 1 shows the results of subgroup analyses based on baseline characteristics. SGLT2-i and RAS-i combination therapy was generally associated with reduced risk of ESKD across most subgroups (eFig. 2C). However, patients without dyslipidemia (*p* = 0.009) and those using insulin (< 0.001) appeared to benefit more from RAS-i therapy alone and patients with dyslipidemia (*p* = 0.009) appear to benefit more from SGLT2-i therapy alone (eFig. 2C).

### Supplemental analyses

To minimize potential selection bias, we conducted sensitivity analyses using alternative populations: one including individuals on two or more antidiabetic medications, and another including individuals on two or more antidiabetic medications while excluding those using other antihypertensive medications among RAS-i users (eTables 1 and 2). The results were consistent with the main analysis. Because death is a competing risk for ESKD, we assessed the risk of ESKD using a competing risk model that accounted for death (eTable 3). The results were also consistent with the main analysis.

## Discussion

This retrospective, nationwide study suggests that combination therapy with SGLT2 inhibitors and RAS inhibitors may offer greater renal and survival benefits for diabetic patients with hypertension compared to either SGLT2 inhibitors or RAS inhibitors alone, or to those not receiving either therapy. This study first showed that SGLT2 inhibitors alone significantly reduced mortality but did not lower ESKD risk. Also, RAS inhibitors alone modestly reduced mortality but were associated with increased risk of ESKD. This association was consistent across most subgroups, with higher-risk patients, such as those aged 65 or older and those with longer diabetes duration, proteinuria, or prolonged hypertension showing greater benefits from combination therapy. The findings remained robust in sensitivity analyses across other populations. Despite the limitations of this retrospective cohort study, it provides valuable insight into the impact of RAS inhibitors and SGLT2 inhibitors, alone or in combination, on ESKD incidence and all-cause mortality in patients with diabetes and hypertension.

Our study found that SGLT2 inhibitors were associated with reduced risk of ESKD (HR 0.56, 95% CI 0.33–0.95) and all-cause mortality (HR 0.73, 95% CI 0.60–0.89) among RAS inhibitor users. These findings align with the CREDENCE trial, which investigated patients with type 2 diabetes, CKD, and RAS inhibitor use, showing that canagliflozin reduced the risk of ESKD (HR 0.68, 95% CI 0.54–0.86) and all-cause mortality (HR 0.83, 95% CI 0.68–1.02) [15]. Similarly, the DAPA-CKD trial, with 90% of patients using RAS inhibitors, demonstrated that dapagliflozin lowered the risk of ESKD (HR 0.64, 95% CI 0.50–0.82) and all-cause mortality (HR 0.69, 95% CI 0.53–0.88) [16]. The EMPA-KIDNEY trial, with 80% of patients using RAS inhibitors, reported consistent results, with empagliflozin reducing the risk of ESKD (HR 0.67, 95% CI 0.52–0.85) and all-cause mortality (HR 0.87, 95% CI 0.70–1.08) [17]. This indicated that combination therapy may provide the greatest renal and survival benefits for diabetic patients with hypertension. Additionally, the results of subgroup analysis, that higher-risk patients such as those aged 65 or older and those with longer diabetes duration, proteinuria, or prolonged hypertension showed greater benefit from combination therapy, also supported the advantages of combination therapy.

Notably, SGLT2 inhibitors alone did not reduce ESKD risk in our study. The renal protective effects of SGLT2 inhibitors have been attributed to multiple mechanisms, including hemodynamic, metabolic, and anti-inflammatory pathways [21]. The key mechanism is the restoration of tubuloglomerular feedback, which helps regulate renal hemodynamics and contributes to kidney protection [21]. Glomerular hyperfiltration, an early hemodynamic change, is believed to accelerate kidney function decline by increasing glomerular pressure and shear stress [22]. Initiation of SGLT2 inhibitors typically leads to an initial 3–5 mL/min per 1.73 m^2^ decline in eGFR among individuals with eGFR > 60 mL/min per 1.73 m^2^, even after a single dose [23]. While this early eGFR dip may raise concerns, evidence from SGLT2 inhibitor trials consistently shows that this change is reversible and not harmful and instead predicts long-term renal benefits [24]. In the EMPA-REG OUTCOME trial, patients receiving empagliflozin with background RAS blockade experienced a greater acute eGFR dip than those taking empagliflozin alone [14]. Similarly, in patients with type 2 diabetes, the combination of losartan and empagliflozin led to a greater reduction in measured GFR than either treatment alone [25]. These findings suggest an interaction between RAS blockade and SGLT2 inhibitors that enhances hemodynamic effects within the glomerular microcirculation. By reducing glomerular hypertension, their combination is likely to provide broad kidney protection across the CKD spectrum. Our study supports this explanation, reinforcing the synergistic benefits of combining SGLT2 inhibitors with RAS inhibitors in diabetic patients.

Several clinical trials have demonstrated the renoprotective effects of RAS inhibitors in diabetic patients with hypertension [26–28]. A recent meta-analysis of 119 randomized trials in CKD patients with and without diabetes found that RAS inhibitors reduced kidney failure risk [29]. Additionally, ACE inhibitors—but not ARBs—significantly reduced all-cause mortality compared to active controls (OR 0.72, 95% CI 0.53–0.92) [29]. However, in our study, RAS inhibitors alone showed only a modest mortality benefit and did not reduce ESKD risk. One possible explanation for this discrepancy is the suboptimal dosing of RAS inhibitors in real-world clinical practice. Unlike clinical trials, where maximum tolerated doses of RAS inhibitors were consistently used, many patients in routine care receive lower-than-recommended doses due to concerns over rising serum creatinine levels. According to our separate analysis using the NHIS–National Sample Cohort, only 20–30% of patients were prescribed high-dose RAS inhibitors (eTable 4). In contrast, in randomized controlled trials, 71% of patients in the losartan group achieved and maintained a 100-mg daily dose in the RENAAL trial, and 83% did so in the IDNT trial [26, 28]. Furthermore, a meta-analysis demonstrated a dose-dependent renoprotective effect of RAS inhibitors [30]. Clinical trials have demonstrated that only full-dose ACE inhibitors and ARBs effectively slow kidney disease progression, whereas subtherapeutic doses offer limited benefit [3]. In line with this, the American Diabetes Association recommends maximizing the dose of either an ACE inhibitor or an ARB in patients with diabetes, hypertension, and albuminuria to prevent CKD progression [3]. Given these findings, further research is needed to explore the discrepancy between RCTs and real-world findings.

The strength of our study lies in the use of a large-scale nationwide database representing the entire Korean population. Also, this was the first study to evaluate the renal protective effects of SGLT2 inhibitors alone without RAS inhibitors. However, this study has certain limitations. First, as this was a retrospective and observational study, selection bias was unavoidable. We compared patient groups with a similar number or pattern of prescribed diabetes and hypertension medications to minimize selection bias. However, to account for the potential introduction of other forms of selection bias resulting from this approach, we conducted sensitivity analyses with different populations, but the results were similar. Second, as a retrospective, observational study, some unidentified factors could have affected the results, although the analyses were conducted after adjusting for most of the available demographic and clinical factors. Third, the RAS inhibitor alone group faced higher risk of ESKD in our study, and we explained this due to the suboptimal dosing of RAS inhibitors in real-world clinical practice. However, we could not confirm this due to strict NHID data policies that did not provide information on RAS inhibitor dosage. Fourth, we defined comorbidities such as diabetes, hypertension, and dyslipidemia using claims data. While this method may not be perfectly accurate, we enhanced precision by creating operational definitions that combined diagnosis, blood glucose, blood pressure, lipid profile, and prescription records. Fifth, owing to the retrospective nature of this study, causality could not be inferred. However, to minimize the likelihood of reverse causation, we excluded individuals with a history of ESKD and adopted a one-year lag period. Finally, the study’s generalizability to other ethnicities may be limited since it focused on the Korean NHID.

## Conclusions

This nationwide cohort study of patients with diabetes and hypertension showed that RAS inhibitors and SGLT2 inhibitors together provide the greatest renal and survival benefits in patients with diabetes and hypertension. SGLT2 inhibitors alone significantly reduce mortality, while RAS inhibitors alone have a modest impact and may not lower ESKD risk.

## Supplementary Information

Additional file1.

## Data Availability

The data that support the findings of this study are available from the National Health Insurance Sharing Service (NHISS, https://nhiss.nhis.or.kr/). However, restrictions apply regarding the availability of the data, which were used with permission for the present study, and are therefore not publicly available. However, they may be made available through the corresponding author, upon reasonable request and with permission from the NHIS.

## Abbreviations

ACE: Angiotensin-converting enzyme
ARB: Angiotensin receptor blocker
BP: Blood pressure
CI: Confidence interval
CKD: Chronic kidney disease
DKD: Diabetic kidney disease
ESKD: End-stage kidney disease
GFR: Glomerular filtration rate
HDL: High-density lipoprotein
HR: Hazard ratios
LDL: Low-density lipoprotein
NHID: National health information database
NHIS: National health insurance service
RAS: Renin–angiotensin–aldosterone system
SD: Standard deviation
SGLT2: Sodium-glucose cotransporter 2
TG: Triglycerides
