# Supplementary material for: Effects of RAS and SGLT2 inhibitors alone or in combination on end-stage kidney disease and/or all-cause death in patients with both diabetes and hypertension: a nationwide cohort study
Source: Cardiovasc Diabetol. 2025 Jul 14;24:288. doi: 10.1186/s12933-025-02846-x (PMC12257671; doi:10.1186/s12933-025-02846-x)

eTable 1. Number, incidence rate, and hazard ratio of end-stage kidney disease in patients on two or more medications for diabetes.

|  | Number of patients | Number of events | Duration  (person-years) | Rate* | Hazard ratio (95% confidence interval) | | | | |
| --- | --- | --- | --- | --- | --- | --- | --- | --- | --- |
|  |  |  |  |  | Unajusted | Model 1 | Model 2 | Stratified by the use of | |
|  |  |  |  |  |  |  |  | SGLT2-i | RAS-i |
| **ESKD or all-cause death** | | | | | | | | | |
| SGLT2-i (-) & RAS (-) | 165916 | 15806 | 885451 | 17.9 | 1 (ref.) | 1 (ref.) | 1 (ref.) | 1 (ref.) | 1 (ref.) |
| SGLT2-i (+) & RAS-i (-) | 5191 | 174 | 25315 | 6.9 | 0.37 (0.32, 0.43) | 0.65 (0.56, 0.76) | 0.67 (0.58, 0.78) | 0.67 (0.58, 0.78) | 1 (ref.) |
| SGLT2-i (-) & RAS-i (+) | 500718 | 58652 | 2704461 | 21.7 | 1.22 (1.20, 1.25) | 1.19 (1.17, 1.21) | 1.08 (1.06, 1.10) | 1 (ref.) | 1.08 (1.06, 1.10) |
| SGLT2-i (+) & RAS-i (+) | 15157 | 795 | 75163 | 10.6 | 0.57 (0.53, 0.61) | 0.89 (0.83, 0.96) | 0.85 (0.79, 0.91) | 0.78 (0.73, 0.84) | 1.25 (1.06, 1.48) |
| **ESKD** |  |  |  |  |  |  |  |  |  |
| SGLT2-i (-) & RAS (-) | 165916 | 1531 | 885451 | 1.7 | 1 (ref.) | 1 (ref.) | 1 (ref.) | 1 (ref.) | 1 (ref.) |
| SGLT2-i (+) & RAS-i (-) | 5191 | 26 | 25315 | 1.0 | 0.61 (0.41, 0.90) | 0.65 (0.44, 0.96) | 0.94 (0.64, 1.39) | 0.94 (0.64, 1.39) | 1 (ref.) |
| SGLT2-i (-) & RAS-i (+) | 500718 | 12437 | 2704461 | 4.6 | 2.65 (2.52, 2.80) | 2.55 (2.42, 2.69) | 1.68 (1.59, 1.77) | 1 (ref.) | 1.68 (1.59, 1.77) |
| SGLT2-i (+) & RAS-i (+) | 15157 | 166 | 75163 | 2.2 | 1.30 (1.10, 1.52) | 1.32 (1.13, 1.55) | 1.22 (1.03, 1.43) | 0.72 (0.62, 0.84) | 1.29 (0.85, 1.95) |
| **All-cause death** | |  |  |  |  |  |  |  |  |
| SGLT2-i (-) & RAS (-) | 165916 | 14621 | 888697 | 16.5 | 1 (ref.) | 1 (ref.) | 1 (ref.) | 1 (ref.) | 1 (ref.) |
| SGLT2-i (+) & RAS-i (-) | 5191 | 149 | 25364 | 5.9 | 0.34 (0.29, 0.40) | 0.67 (0.57, 0.79) | 0.67 (0.57, 0.79) | 0.67 (0.57, 0.79) | 1 (ref.) |
| SGLT2-i (-) & RAS-i (+) | 500718 | 48746 | 2733117 | 17.8 | 1.10 (1.08, 1.12) | 1.07 (1.05, 1.10) | 1.02 (1.00, 1.04) | 1 (ref.) | 1.02 (1.00, 1.04) |
| SGLT2-i (+) & RAS-i (+) | 15157 | 652 | 75468 | 8.6 | 0.50 (0.46, 0.54) | 0.86 (0.80, 0.93) | 0.83 (0.76, 0.89) | 0.81 (0.75, 0.87) | 1.23 (1.03, 1.47) |

*Rate: events per 1000 person-years.

Model 1: adjusted by age and sex.

Model 2: adjusted by age, sex, household income, smoking status, alcohol drinking, regular exercise, body mass index, systolic blood pressure, glucose, eGFR, duration of diabetes, duration of hypertension, diabetes medication, and hypertension medication.

eTable 2. Number, incidence rate, and hazard ratio of end-stage kidney disease in patients on two or more medications for diabetes while excluding those using other antihypertensive medications among RAS-i users.

|  | Number of patients | Number of events | Duration  (person-years) | Rate* | Hazard ratio (95% confidence interval) | | | | |
| --- | --- | --- | --- | --- | --- | --- | --- | --- | --- |
|  |  |  |  |  | Unajusted | Model 1 | Model 2 | Stratified by the use of | |
|  |  |  |  |  |  |  |  | SGLT2-i | RAS-i |
| **ESKD or all cause death** | | | | | | | | | |
| SGLT2-i (-) & RAS (-) | 165916 | 15806 | 885451 | 17.9 | 1 (ref.) | 1 (ref.) | 1 (ref.) | 1 (ref.) | 1 (ref.) |
| SGLT2-i (+) & RAS-i (-) | 5191 | 174 | 25315 | 6.9 | 0.37 (0.32, 0.43) | 0.69 (0.60, 0.80) | 0.72 (0.62, 0.83) | 0.72(0.62, 0.83) | 1 (ref.) |
| SGLT2-i (-) & RAS-i (+) | 109573 | 10063 | 598885 | 16.8 | 0.95 (0.93, 0.97) | 1.02 (0.99, 1.04) | 0.93 (0.90, 0.95) | 1 (ref.) | 0.93 (0.90, 0.95) |
| SGLT2-i (+) & RAS-i (+) | 3177 | 110 | 15861 | 6.9 | 0.37 (0.31, 0.45) | 0.71 (0.59, 0.85) | 0.66 (0.54, 0.79) | 0.71(0.59, 0.86) | 0.92 (0.72, 1.16) |
| **ESKD** |  |  |  |  |  |  |  |  |  |
| SGLT2-i (-) & RAS (-) | 165916 | 1531 | 885451 | 1.7 | 1 (ref.) | 1 (ref.) | 1 (ref.) | 1 (ref.) | 1 (ref.) |
| SGLT2-i (+) & RAS-i (-) | 5191 | 26 | 25315 | 1.0 | 0.61 (0.41, 0.90) | 0.68 (0.46, 1.00) | 1.01 (0.68, 1.49) | 1.01 (0.68, 1.49) | 1 (ref.) |
| SGLT2-i (-) & RAS-i (+) | 109573 | 1503 | 598885 | 2.5 | 1.44 (1.35, 1.55) | 1.41 (1.31, 1.51) | 0.99 (0.92, 1.07) | 1 (ref.) | 0.99 (0.92, 1.07) |
| SGLT2-i (+) & RAS-i (+) | 3177 | 14 | 15861 | 0.9 | 0.52 (0.31, 0.88) | 0.57 (0.34, 0.96) | 0.56 (0.33, 0.96) | 0.57 (0.34, 0.97) | 0.56 (0.29, 1.07) |
| **All-cause death** | |  |  |  |  |  |  |  |  |
| SGLT2-i (-) & RAS (-) | 165916 | 14621 | 888697 | 16.5 | 1 (ref.) | 1 (ref.) | 1 (ref.) | 1 (ref.) | 1 (ref.) |
| SGLT2-i (+) & RAS-i (-) | 5191 | 149 | 25364 | 5.9 | 0.34 (0.29, 0.40) | 0.68 (0.58, 0.80) | 0.69 (0.59, 0.81) | 0.69 (0.59, 0.81) | 1 (ref.) |
| SGLT2-i (-) & RAS-i (+) | 109573 | 8857 | 602146 | 14.7 | 0.90 (0.88, 0.93) | 0.98 (0.96, 1.01) | 0.92 (0.89, 0.94) | 1 (ref.) | 0.92 (0.89, 0.94) |
| SGLT2-i (+) & RAS-i (+) | 3177 | 97 | 15880 | 6.1 | 0.35 (0.29, 0.43) | 0.72 (0.59, 0.88) | 0.67 (0.55, 0.82) | 0.73 (0.60, 0.89) | 0.97 (0.75, 1.25) |

* Rate: events per 1000 person-years.

Model 1: adjusted by age and sex.

Model 2: age, sex, household income, smoking status, alcohol drinking, regular exercise, body mass index, systolic blood pressure, glucose, eGFR, duration of diabetes, duration of hypertension, diabetes medication, hypertension medication.

eTable 3. Number, incidence rate, and hazard ratio of end-stage kidney disease in patients using a competing risk model that accounted for death.

|  | Number of patients | Number of events | Competing event | Duration  (person-years) | Rate* | Hazard ratio (95% confidence interval) | | | | |
| --- | --- | --- | --- | --- | --- | --- | --- | --- | --- | --- |
|  |  |  |  |  |  | Unajusted | Model 1 | Model 2 | Stratified by the use of | |
|  |  |  |  |  |  |  |  |  | SGLT2-i | RAS-i |
| **ESKD** |  |  |  |  |  |  |  |  |  |  |
| SGLT2-i (-) & RAS (-) | 144227 | 1135 | 11552 | 807284 | 1.4 | 1 (ref.) | 1 (ref.) | 1 (ref.) | 1 (ref.) | 1 (ref.) |
| SGLT2-i (+) & RAS-i (-) | 4806 | 22 | 127 | 23801 | 0.9 | 0.67  (0.44, 1.02) | 0.72  (0.47, 1.09) | 1.06  (0.67, 1.61) | 1.06  (0.70, 1.61) | 1 (ref.) |
| SGLT2-i (-) & RAS-i (+) | 109573 | 1503 | 8560 | 627379 | 2.4 | 1.70  (1.57, 1.83) | 1.65  (1.53, 1.79) | 1.17  (1.07, 1.27) | 1 (ref.) | 1.17  (1.07, 1.27) |
| SGLT2-i (+) & RAS-i (+) | 3177 | 14 | 96 | 16157 | 0.9 | 0.62  (0.37, 1.06) | 0.65  (0.38, 1.10) | 0.63  (0.37, 1.06) | 0.54  (0.32, 0.91) | 0.60  (0.31, 1.16) |

eTable 4. Distribution of RAS inhibitors Dosage Among Users from the NHIS–National Sample Cohort.

| year | **2009** | **2010** | | **2011** | | **2012** | | **2013** | | **2014** | | **2015** | | **2016** | | **2017** | |
| --- | --- | --- | --- | --- | --- | --- | --- | --- | --- | --- | --- | --- | --- | --- | --- | --- | --- |
| **Simalr population of our main analysis** | | | | | | | | | | | | | | | | | |
| **RAS inhibitor dosage** | | |  | |  | |  | |  | |  | |  | |  | |  |
| low | 8 (2.44) | 1 (0.38) | | 4 (3.17) | | 2 (2) | | 3 (3.45) | | 4 (4.71) | | 8 (9.3) | | 10 (13.16) | | 12 (15.79) | |
| moderate | 244 (74.39) | 195 (73.58) | | 97 (76.98) | | 62 (62) | | 65 (74.71) | | 67 (78.82) | | 59 (68.6) | | 53 (69.74) | | 58 (76.32) | |
| high | 76 (23.17) | 69 (26.04) | | 25 (19.84) | | 36 (36) | | 19 (21.84) | | 14 (16.47) | | 19 (22.09) | | 13 (17.11) | | 6 (7.89) | |
| **RAS inhibitor use among pairents with diabetes** | | | | | | | | | | | | | | | | | |
| **RAS inhibitor dosage** | | | | | | | | | | | | | | | | | |
| low | 112 (2.16) | 71 (1.54) | | 42 (1.86) | | 24 (1.24) | | 35 (2.25) | | 43 (3.12) | | 34 (2.41) | | 51 (3.45) | | 69 (4.57) | |
| moderate | 3531 (68.23) | 3213 (69.64) | | 1569 (69.49) | | 1343 (69.26) | | 1095 (70.42) | | 974 (70.63) | | 975 (69.1) | | 1037 (70.07) | | 1054 (69.76) | |
| high | 1532 (29.6) | 1330 (28.83) | | 647 (28.65) | | 572 (29.5) | | 425 (27.33) | | 362 (26.25) | | 402 (28.49) | | 392 (26.49) | | 388 (25.68) | |

Low, moderate, and high are definded according to table below.

| RAS blocker | Low Dose | Moderate Dose | High Dose |
| --- | --- | --- | --- |
| Losartan | 25 mg/day | 50 mg/day | 100 mg/day |
| Valsartan | 40–80 mg/day | 160 mg/day | 320 mg/day |
| Candesartan | 4 mg/day | 8 mg/day | 16-32 mg/day |
| Telmisartan | 20 mg/day | 40 mg/day | 80 mg/day |
| Olmesartan | 10 mg/day | 20 mg/day | 40 mg/day |
| Irbesartan | 75 mg/day | 150 mg/day | 300 mg/day |
| Ramipril | 2.5 mg/day | 5 mg/day | 10 mg/day |
| Enalapril | 5 mg/day | 10 mg/day | 20 mg/day |
| Lisinopril | 5 mg/day | 10 mg/day | 20–40 mg/day |
| Perindopril | 2 mg/day | 4 mg/day | 8 mg/day |

eFigure 1. The cumulative incidence of end-stage kidney disease (ESKD) and/or all-cause death based on Kaplan–Meier analysis. The P-value was calculated using the log-rank test.


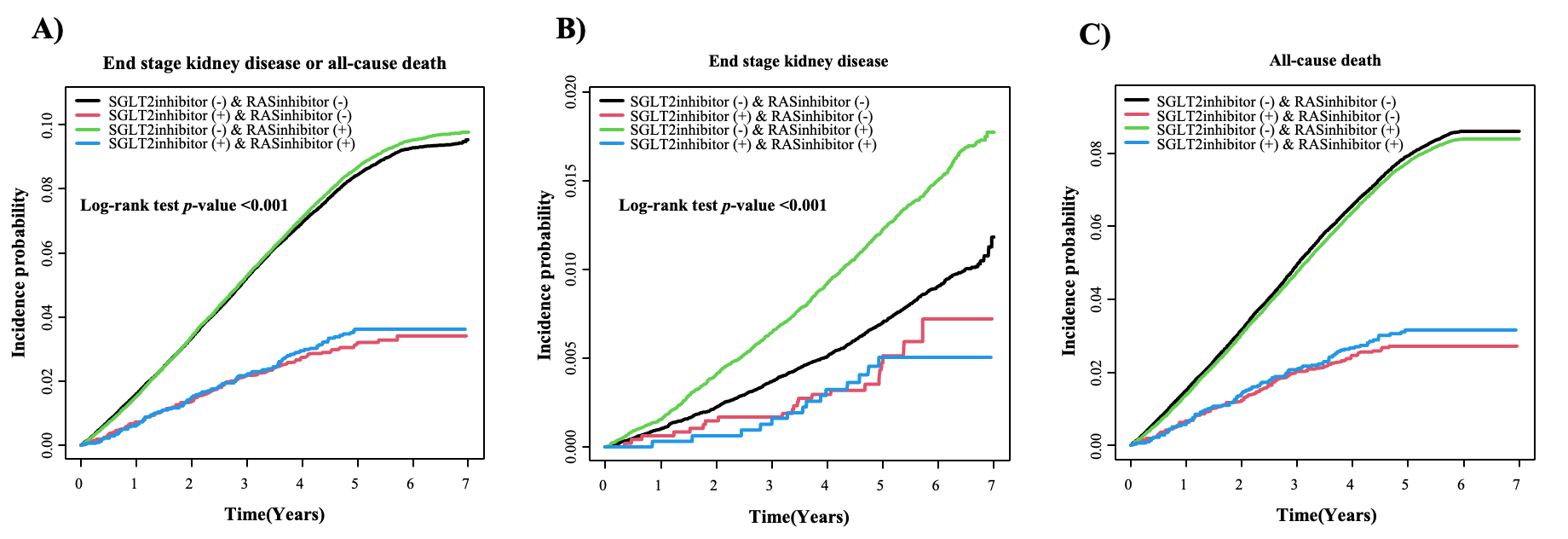


eFigure 2. Hazard ratios for outcomes between groups. Subjects are organized into pre-specified subgroups adjusted for age, sex, body mass index, dyslipidemia, diabetes duration, hypertension duration, insulin usage, number of diabetes medication, eGFR, and proteinuria.

1. All-cause death or ESKD


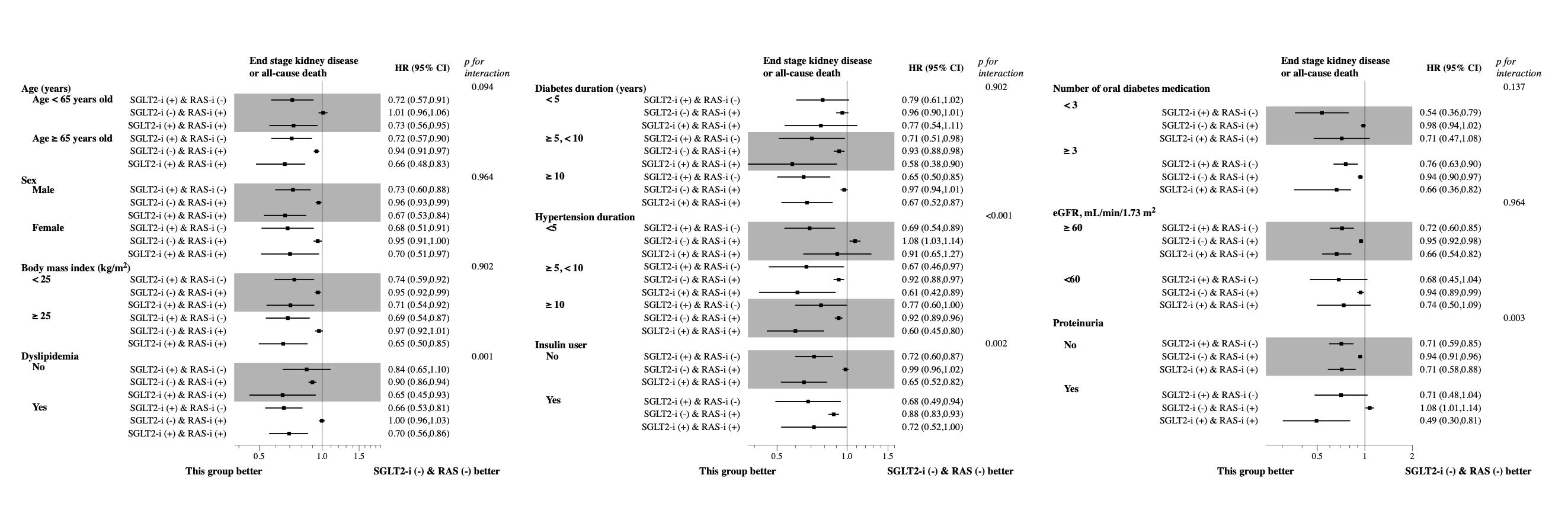


1. ESKD


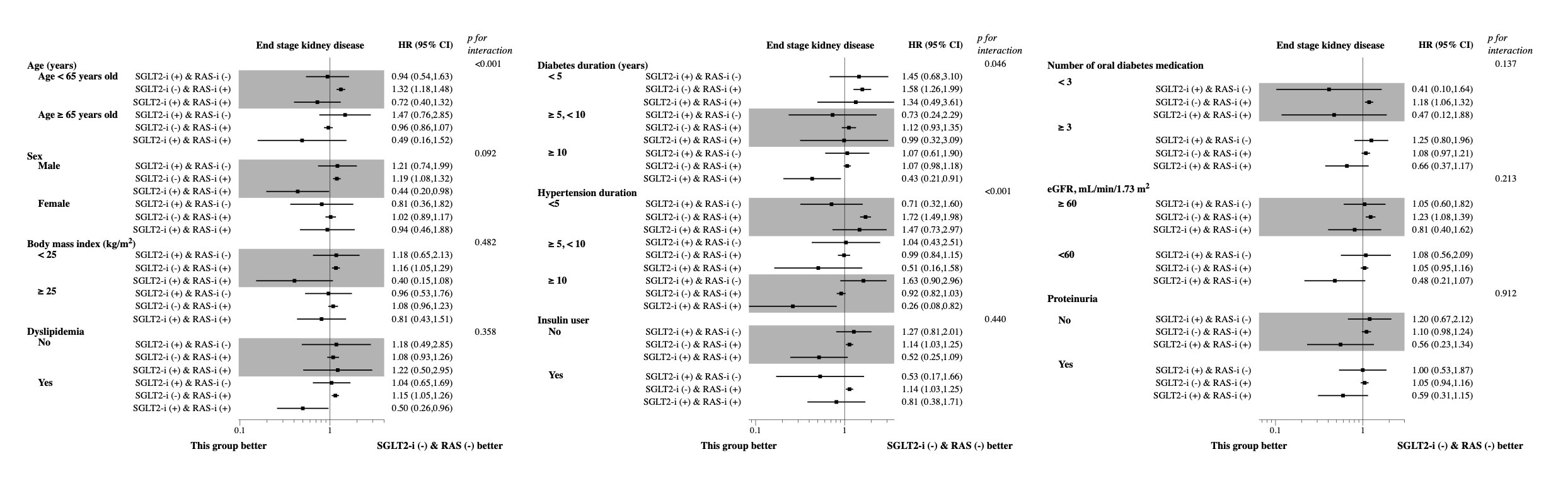


1. All-cause death


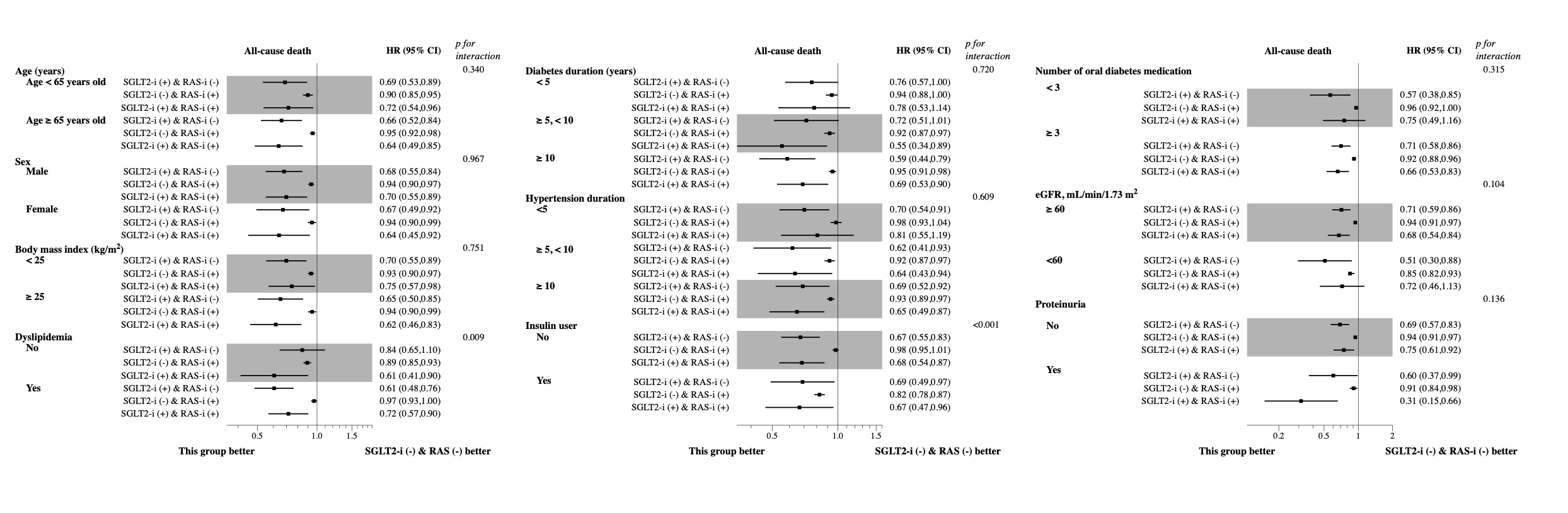

Supplement: Supplementary file 1 — Additional file1. [file 12933_2025_2846_MOESM1_ESM.docx]
